# Profiling ultra-processed foods in Thailand: sales trend, consumer expenditure and nutritional quality

**DOI:** 10.1186/s12992-023-00966-1

**Published:** 2023-08-31

**Authors:** Sirinya Phulkerd, Natjera Thongcharoenchupong, Sarah Dickie, Priscila Machado, Julie Woods, Ladda Mo-Suwan, Piyada Prasertsom, Chantana Ungchusak, Chiraporn Khitdee, Mark Lawrence

**Affiliations:** 1 Institute for Population and Social Research, Mahidol University, Phutthamonthon, Nakhon Pathom, Thailand; 2 Institute for Physical Activity and Nutrition, School of Exercise and Nutrition Sciences, Deakin University, Geelong, VIC Australia; 3 Department of Paediatrics, Faculty of Medicine, Prince of Songkla University, Hat Yai, Songkhla Thailand; 4 Sweet Enough Network, The Foundation of Oral Health, Mueang, Nonthaburi, Thailand; 5 Healthy Food Plan, Thai Health Promotion Foundation, Sathorn, Bangkok, Thailand; 6 Department of Health, Bureau of Dental Health, Ministry of Public Health, Mueang, Nonthaburi, Thailand

**Keywords:** NOVA food classification system, Ultra-processed foods, Food supply, Diet-related noncommunicable diseases, Thailand, Food policy

## Abstract

**Background:**

Ultra-processed foods (UPF) are associated with adverse health outcomes. This study aimed to analyse the national trends in retail sales, consumer expenditure and nutritional quality of UPFs in Thailand.

**Methods:**

The study used data from the Euromonitor Passport database for analysis of retail sales and consumer expenditure, and from the Mintel Global New Products Database for nutritional analysis using the WHO Southeast Asian Region nutrient profile model.

**Results:**

The study found the highest per capita sales volume and value of UPFs in 2021 were sauces, dressings & condiments (8.4 kg/capita) and carbonated soft drinks (27.1 L/capita), respectively. However, functional & flavoured water, ready-made meals and baked goods had the highest observed (2012–2021) and expected (2021–2026) sales growth. Supermarkets were responsible for most of the UPF sales since 2012, but convenience stores had larger growth in retail values. Growth in consumer expenditure per capita on UPFs from 2012 to 2020, ranged between 12.7% and 34%, and till 2026 is forecast to grow between 26% and 30%. More than half of UPFs exceeded at least one nutrient cutoff, 59.3% for total fats, 24.8% for saturated fats, 68.2% for total sugars and 94.3% for sodium.

**Conclusions:**

The findings suggest a need for regulatory and non-regulatory measures such as UPF taxation and marketing restrictions, and market incentives for producing non-UPFs. A system for regularly monitoring and evaluating healthiness (both nutritional and processing aspects) of food products, especially UPFs, is required.

**Supplementary Information:**

The online version contains supplementary material available at 10.1186/s12992-023-00966-1.

## Background

There has been a rapid industrialisation of food systems around the world and the proliferation of ultra-processed foods (UPF) is a powerful marker of this industrialization [1]. The substantial body of evidence shows that UPFs are associated with adverse health outcomes including cardio-metabolic risks and asthma in children; and obesity, cancer, type-2 diabetes, cardiovascular diseases, irritable bowel syndrome, depression and mortality in adults [2]. An increasing prevalence of non-communicable diseases (NCD) has been reported to be associated with globalisation e.g. exposure to the global mass media, food technology and innovations and modern marketing techniques have modified dietary preferences [3, 4]. Consequently, it led to major changes in the composition of diet in particular a shift of diets towards more high-fat, refined carbohydrate and low-fibre diet.

Increasing dietary exposure to UPFs can also displace nutritious unprocessed and minimally processed foods in the diet, and lead to vitamin and mineral deficiencies [5, 6]. However, UPFs have been associated with adverse health outcomes independently to their specific nutrient content (e.g. salt, sugar and saturated fat) [7]. Other mechanisms such as the degraded food matrix, contaminants from processing (acrylamides and advanced glycation end products) and particular food additives in UPFs (e.g. emulsifiers and artificial sweeteners) were also observed that contribute to those associations [7]. UPF was found to be linked to environmental degradation that can affect health, through for example biodiversity loss, greenhouse gas emissions, land use and food waste [8].

The definition of UPF was developed within the NOVA food classification system which classifies foods and drinks into four groups (unprocessed or minimally processed foods; processed culinary ingredients; processed foods; and UPF), according to the extent and purpose of the industrial processing [9]. Ultra-processed foods are described as formulations of ingredients, mostly of exclusive industrial use, that contain substances extracted from foods (e.g. fructose, hydrogenated oils, protein isolates) and cosmetic additives (colours, flavours, thickeners). Their common characteristics are *“little or no whole foods, are ready-to-consume or heat up, and are fatty, salty or sugary and depleted in dietary fibre, protein, various micronutrients and other bioactive compounds.”* Examples of UPFs include potato crisps, confectionary, sugar-sweetened beverages (SSB), ready-to-heat frozen meals, instant soups and noodles.

Concerns about UPF exposure in population diets has gained much attention globally. For example, sales growth and the poor nutritional quality of UPFs were referenced in a 2016 ‘Foresight’ report published by the Global Panel on Agriculture and Food Systems for Nutrition [10]. More recently, a FAO report describing the global increased sales of UPFs was published [11], and there has been a call from the United Nations Food Systems Summit for governments and global communities to take urgent action on reducing UPF production, distribution and consumption [12]. Additionally, the concept of UPF is being included in national food policies and practices in several countries, for example, the Dietary Guidelines for the Brazilian Population, and other official healthy eating guidelines in countries such as France, Belgium, Canada, Peru, Israel, Ecuador and Uruguay [13]. [5, 6, 8]

There is a rapid transition underway across South East Asia (SEA) towards increasing amounts of UPFs in both the food supply and in dietary intakes [14]. Developments in the retail sector have also contributed to growing and diversifying UPF markets, especially the growth of modern food retail, often dominated by transnational grocery retailers such as Tesco and 7-Eleven or other regional and national players. Convenience stores, supermarkets and hypermarkets in particular, have continued to grow in several SEA countries since 2012 according to Euromonitor data [15].

The UPF concept has not yet been applied in Thailand for the purpose of assessing individual-level food consumption. However, according to the 2017 Food Consumption Behaviour Survey of the National Statistical Office Thailand, processed foods and beverages were commonly consumed food groups among Thai people [16]. SSB, packaged ready-to-eat foods and savoury snacks were most commonly consumed, with 76.2%, 59.3% and 48.3% of the total population consuming these foods, respectively. Overweight and obesity, and diet-related NCDs (such as cardiovascular diseases and diabetes) contribute to a significant proportion of deaths in the Thai population each year. The most recent mortality data report in Thailand (2014) showed that overweight and obesity was the third leading risk factor contributing to mortality in Thailand after hypertension and smoking, accounting for 30,986 deaths (of total 435,624 deaths) [17, 18]. In the same year, cerebrovascular diseases were the number one cause of death for both Thai men and women (approximately 30,000 and 31,000 deaths, respectively), and diabetes caused approximately 12,000 and 19,000 deaths in Thai men and women, respectively [19].

UPF products have generally poor nutritional quality [20, 21]. They often contain excessive amounts of nutrients when consumed, in excess, can be detrimental to population health, in particular free sugars, total fat, saturated fat and sodium, compared with unprocessed or minimally processed foods. According to the analyses in several countries in Latin America and the Caribbean regions, all analysed UPF products contain excess free sugars, total fat, saturated fat and sodium, and importantly, 70% exceeded the recommended level of two or three of these nutrients [21]. Major contributing sources to these nutrients were from SSBs, biscuits, confectionery and industrial breads. UPFs are designed to be convenient and hyper-palatable to consumers [9], and as such, can induce overconsumption. Accordingly, the dietary share of UPFs has been recommended for monitoring and benchmarking population diet quality globally [22]. Such measurements have not been previously performed in Thailand.

It is important for policy makers aiming to encourage healthier dietary choices to have up to date information on UPF availability in the food supply and UPF purchasing behaviours. However, there is currently no such evidence that is easily accessible and used by policy makers in Thailand. Therefore, this study aimed to analyse the national trends in retail sales of UPFs, consumer expenditure on UPFs and the nutritional quality of UPFs sold in Thailand.

## Methods

This study used secondary data on retail sales, consumer expenditure and nutritional information and ingredients of packaged food and beverage products available in the Thailand market. The study applied the concept of UPF as defined by the NOVA food classification according to Monteiro et al. [9]. Criteria for NOVA classification is described in Table A1. Foods and beverages were either classified as UPF or non-UPF (NOVA groups 1–3; unprocessed or minimally processed foods, processed culinary ingredients and processed foods). UPFs were classified based on the presence of one or more industrial food substances (substances of no or rare culinary use) or cosmetic additives in the ingredients list displayed on the food label. Methodology from Monteiro et al. 2019 [9] and Dickie et al. 2023 [23] guided identification of UPF characterising ingredients.

### Data sources

This study used secondary data from two databases, the Euromonitor Passport Database and the Mintel Global New Products Database (Mintel GNPD). Data on retail sales and Thai consumer expenditure between 2012 and 2021 were obtained from Euromonitor Passport database [15] with projections to 2026. Data on product ingredients between 2015 and 2021 were from the Mintel GNPD.

#### Retail sales and consumer expenditure

Information on UPF sales in different retail formats i.e., hypermarkets (retail outlets with a selling space of over 2,500 square metres and with a focus on selling both grocery and non-grocery merchandise), supermarkets, convenience stores, independent small grocers and other grocery retailers (such as kiosks, markets selling predominantly groceries including confectionery tobacco newsagent, and health food stores, food & drink souvenir stores and regional speciality stores), derived from Euromonitor Passport database, was available up to 2021. The sales value and expenditure data were presented in current (nominal) value which includes inflation.

Sales data for the following UPF categories were included: baked goods, breakfast cereals, confectionery & sweet spreads, some dairy products & alternatives, frozen processed potatoes, ice cream & frozen desserts, ready meals, processed meat & seafood, sauces, dressings & condiments, savoury snacks, sweet biscuits, snack bars & fruit snacks,, total ultra-processed beverages, carbonated soft drinks, soft drink concentrates, dairy products & alternatives, functional & flavoured water, juice drinks & nectars, ready-to-drink tea, coffee & Asian speciality drinks, and sports & energy drinks. Category definition for sales data is described in Table A2. However, retail format sales data were not available for margarine and spreads, frozen processed potatoes, instant noodles, meat substitutes, processed meat & seafood, and functional & flavoured water.

Annual consumer expenditure per capita on only certain food and non-alcoholic beverage products was available. This data does not reflect the broad UPF category and that some non-UPFs and UPFs were aggregated and presented together. Euromonitor groups food and beverage categories for consumer expenditure into the following categories: food categories i.e. bread and cereals; meat; fish and seafood; milk, cheese and eggs; oils and fats; fruit; vegetables; sugar and confectionery; and other food, and non-alcoholic beverage categories i.e. coffee, tea and cocoa; and mineral waters, soft drinks, fruit and vegetable juices (Table A3). For the purpose of this study, only the categories determined to mainly consist of UPFs were included for expenditure analysis. The categories of meat; fish and seafood; milk, cheese and eggs; fruit; vegetables; oils and fats; and other food were not included as they include non-UPF items such as fresh meat, fresh fruit, fresh vegetables and culinary herbs.

#### Nutritional classification

This study evaluated the nutritional quality of UPF products sold in the Thailand market in relation to nutrition policy tools that regulate food marketing to children and provision of food in school settings using the Mintel GNPD. As the current version of the nutrient profile model used in Thailand remains under review and consultation, this study used the World Health Organisation Southeast Asian Region (WHO SEA) nutrient profile model for nutritional quality evaluation. This model was adopted by SEA Member States to assess eligibility for marketing of food and non-alcoholic beverages to children and for other purposes of food and nutrition policies such as provision of healthy options in schools [24]. Foods were initially classified using the NOVA classification system and the WHO SEA criteria were then applied to identify UPF products which contain excessive, total fat, saturated fat, free sugars and sodium. The WHO SEA model consists of 18 categories. Three of these categories: fresh and frozen fruits and vegetables, and legumes; fats and oils, and fat emulsions; and fresh and frozen meat, poultry, game, fish and seafood were excluded as they were considered as non-UPF. Therefore, this study grouped UPFs into 15 WHO SEA categories (of total 18 categories) (Table 1) and compared across all selected nutrients.

The average content of these nutrients in each food was calculated, and products were classified as “excessive” if their nutrient content was higher than the criteria summarised in Table 1. A product which did not exceed any of the criteria (on a per 100 g/ml basis) was considered eligible for marketing according to the WHO SEA. All foods from the Mintel GNPD were categorised against the WHO SEA categories and then total fat, saturated fat, total sugars and sodium density were determined.

**Table 1:** Criteria for excessive fat, sugars and sodium (per 100 g/ml) defined by the WHO SEA Nutrient Profile Model

Food category	Total fat (g)	Saturated fat (g)	Total sugars (g)	Added sugars (g)	Sodium (g)	Energy (kcal)
1. **Confectionery**	> 8.0	N	> 6.0	N	N	> 230
2. **Fine bakery wares**	> 8.0	N	> 6.0	N	> 0.25	> 230
3. **Bread and ordinary bakery wares**	> 8.0	N	> 6.0	N	> 0.25	N
4. **Cereals**	> 12.0	N	> 9.0	N	> 0.35	N
5. **Ready-to-eat savouries (savoury snack foods)**						
(a) Potato, cereal or starch-based (from roots, tuber, or legumes) and animal based (from skin)	> 8.0	N	N	> 0.0	> 0.25	> 230
(b) Processed nuts	N	N	N	> 0.0	> 0.5	N
(c) Fish-based	N	N	> 6.0	N	> 0.25	> 230
6. **Beverages**						
(a) Juices	N	N	> 6.0	> 0.0	N	N
(b) Milk and dairy based drinks	> 7.0	N	N	> 0.0	N	N
(c) Water-based flavoured drink	N	N	> 2.0	N	> 0.30	N
(d) Coffee, coffee substitutes, tea, herbal infusions	N	N	> 2.0	N	N	N
(e) Cereal, grain, tree nut-based beverages	N	N	> 6.0	N	> 0.20	N
7. **Frozen dairy based desserts and edible ices**	> 8.0	N	> 12.0	N	> 0.10	> 230
8. **Curded dairy based desserts**	> 7.0	N	> 6.0	N	> 0.10	> 230
9. **Cheese and analogues**	> 20.0	N	N	> 0.0	> 0.60	N
10. **Composite foods (prepared foods)**	> 8.0	> 3.5	> 9.0	N	> 0.35	N
11. **Pasta and noodles and like products**	> 3.0	N	N	N	> 0.25	N
12. **Processed meat, poultry, game, fish and fish products**						
(a) Processed meat, poultry and game products	> 8.0	N	N	N	> 0.40	N
(b) Processed fish and seafood products	> 8.0	> 3.0	N	N	> 0.40	N
13. **Processed fruits and vegetables**	N	N	N	> 0.0	> 0.40	N
14. **Solid-form soybean products**	> 12.0	N	> 5.0	> 0.0	> 0.10	N
15. **Sauces dips, and dressings**	> 12.0	N	> 10.0	N	> 0.30	N

N - No threshold provided

### Data analysis

The analyses were conducted using STATA version 16. The analysis of trends in average annual retail sales of UPFs in Thailand (both volume in tonnes and values in THB million) was undertaken using methods as previously reported by Baker et al. [14]. UPFs were divided into ultra-processed *foods* and ultra-processed *beverages*, and per capita sales volumes were estimated for the categories for all available years.

Time trends in volume sales and consumer expenditure of UPFs were calculated and contributions of food categories to sales and expenditures determined. Descriptive statistics including the frequency and percentage of sales and expenditures of UPF items and their nutritional quality by categories evaluated using the WHO SEA model criteria were calculated. Comparison of UPF items with and without excessive nutrient contents was performed. The data were analysed and the food classification conducted by S.P. and N.T., and independently assessed by other researchers (S.D., P.M., J.W. and M.L.).

## Results

### Retail sales of UPFs

Table 2 presents per capita sales volumes for UPFs in 2021. The highest per capita UPF sale volumes were observed in carbonated soft drinks (27.1 L/capita), sauces, dressings & condiments (8.4 kg/capita), (11.1 kg/capita), and sports & energy drinks (7.4 L/capita), respectively. The highest per capita UPF sale values were found dairy products & alternatives (995.6 THB), sauces, dressings & condiments (687.9 THB), savoury snacks (584.2 THB), and baked goods (542.4 THB), respectively.

**Table 2:** Ultra-processed foods and beverage sales per capita in 2021

Categories	Sale volume per capita (kilogram or litres)	Sale value per capita (THB)
**Ultra-processed foods**		
Baked goods	3.9	542.4
Breakfast cereals	0.1	39
Confectionery & sweet spreads	1.3	368.6
Dairy products	5.5	309.4
Diary alternatives	n/a	n/a
Processed fruit and vegetables	0.8	32.5
Ice cream & frozen desserts	0.9	166.1
Processed meat & seafood	1.7	339.7
Ready meals	1.5	293.6
Sauces, dressings & condiments	8.4	687.9
Savoury snacks	1.4	584.2
Sweet biscuits, snack bars & fruit snacks	0.8	207.8
**Ultra-processed beverages**		
Carbonated soft drinks	27.1	27.1
Soft drink concentrates	0.1	34.1
Dairy products	15.1	747.4
Diary alternatives	5.7	235.2
Functional & flavoured water	n/a	n/a
Juice drinks & nectars	4.6	295
RTD tea, coffee & Asian specialty drinks	8	415.8
Sports & energy drinks	7.4	471

n/a – data not available

**Fig. 1:**
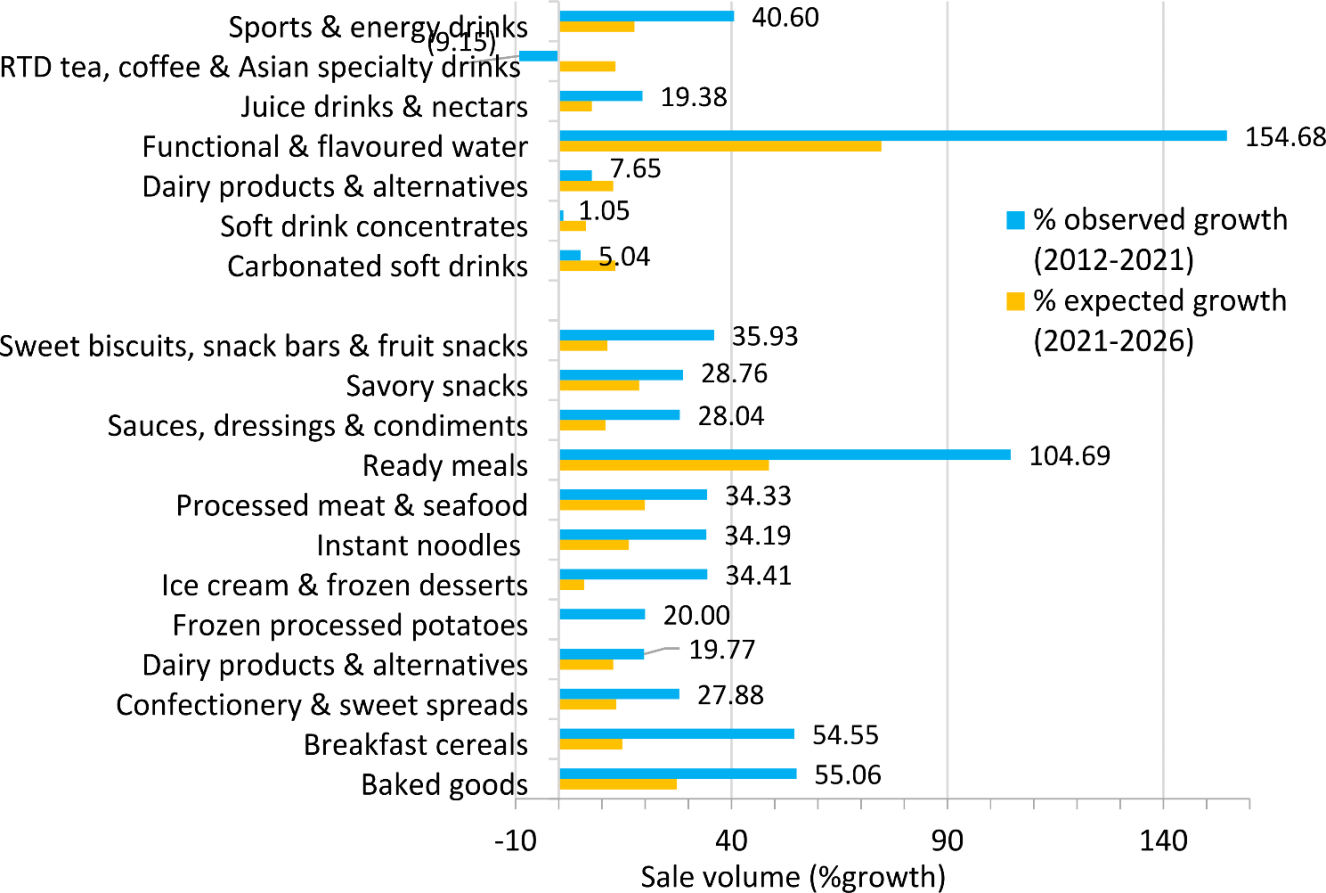
Percent growth of total sales volumes of UPFs in Thailand, 2012–2021, and projections from 2021 to 2026, classified into different UPF categories

**Fig. 2:**
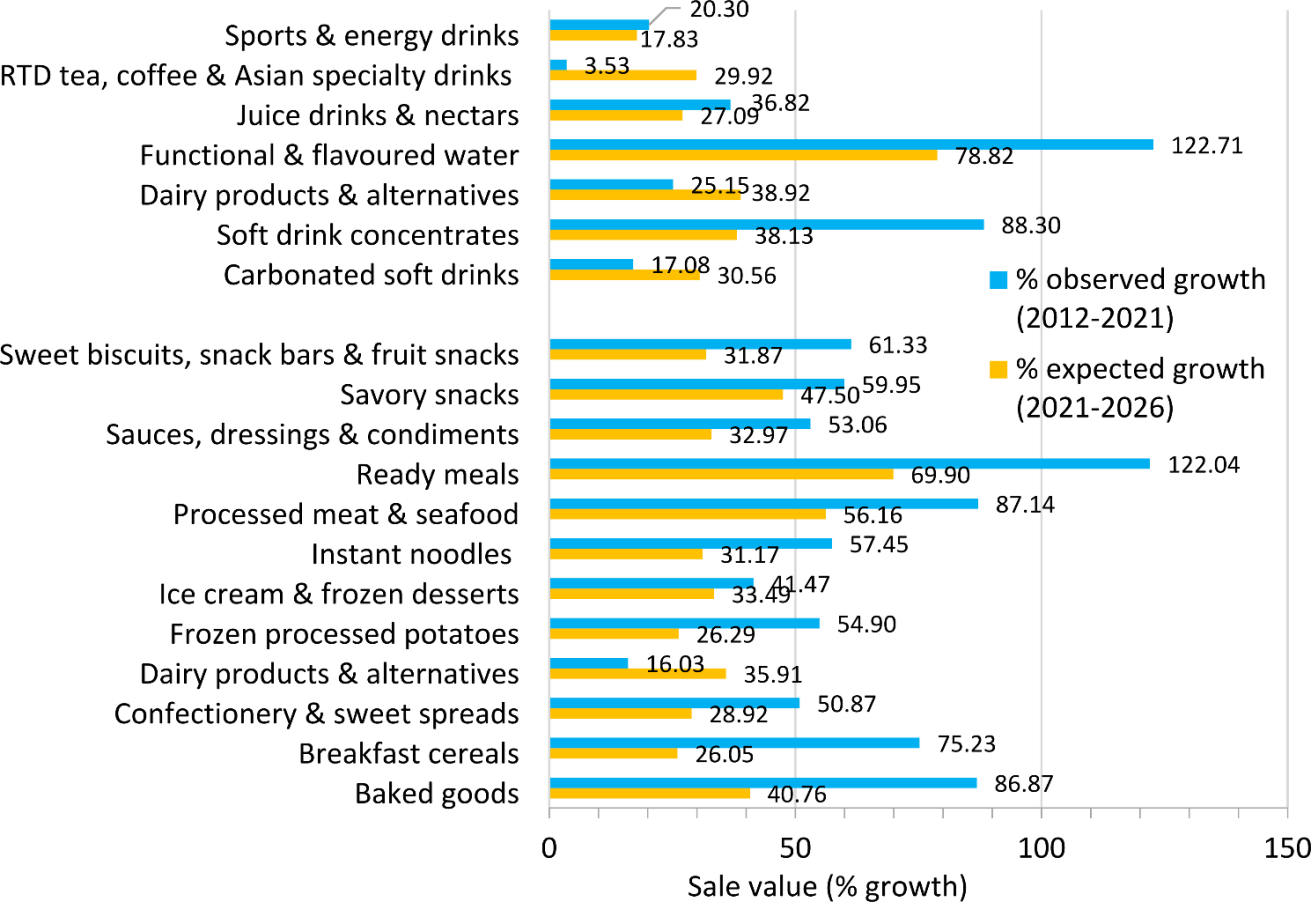
Percent growth of total sales values of UPFs in Thailand, 2012–2021, and projections from 2021 to 2026, classified into different UPF categories

Figures 1 and 2 present overall growth of sales volumes and values of UPFs in Thailand, from 2012 to 2021, and projections from 2021 to 2026.

As presented in Fig. 1, an increase in total sale volumes across UPF categories from 2012 to 2021 was observed ranging from 1.1 to 154.7%. The only category in which sales dropped was ready-to-drink (RTD) tea, coffee & Asian specialty drinks (-9.2%). Specific UPF categories where sale volumes grew fastest were functional & flavoured water (154.7%), followed by ready meals (104.7%), baked goods (55.1%), breakfast cereals (54.6%), and sports & energy drinks (40.1%).

Total sale volumes of all the categories (except frozen processed potatoes) were projected to grow between 5.9% and 74.7% by 2026. Sales of the categories which were projected to grow rapidly were functional & flavoured water (74.7%), followed by ready meals (48.7%), baked goods (27.4%), processed meat & seafood (20%), and processed meat and seafood (20%).

Figure 2 presents the sale values showing that the UPF market grew in all categories between 2012 and 2021. The category where sale values increased least was RTD tea, coffee & Asian specialty drinks (3.5%). Specific UPF categories where sale values grew the most were functional & flavoured water (122.7%), followed by ready meals (122%), soft drink concentrates (88.3%), processed meat & seafood (87.1%), baked goods (86.9%), and breakfast cereals (75.2%).

From 2021 to 2026, the sale values are expected to grow in all food categories. Sales of the UPF categories which are projected to grow by more than 50% are functional & flavoured water (78.8%), followed by ready meals (69.9%), and processed meat and seafood (56.2%). Carbonated soft drinks and RTD tea, coffee & Asian specialty drinks categories are projected to have a higher growth rate when compared to the rate in the last ten years. The carbonated soft drinks category are expected to grow 30.6% by 2026, which almost doubles growth of the sale values in the past ten years (17.1%). Projected growth of the RTD tea, coffee & Asian specialty drinks category is 29.9% which is almost 10 times growth of the past ten years (3.5%).

**Fig. 3:**
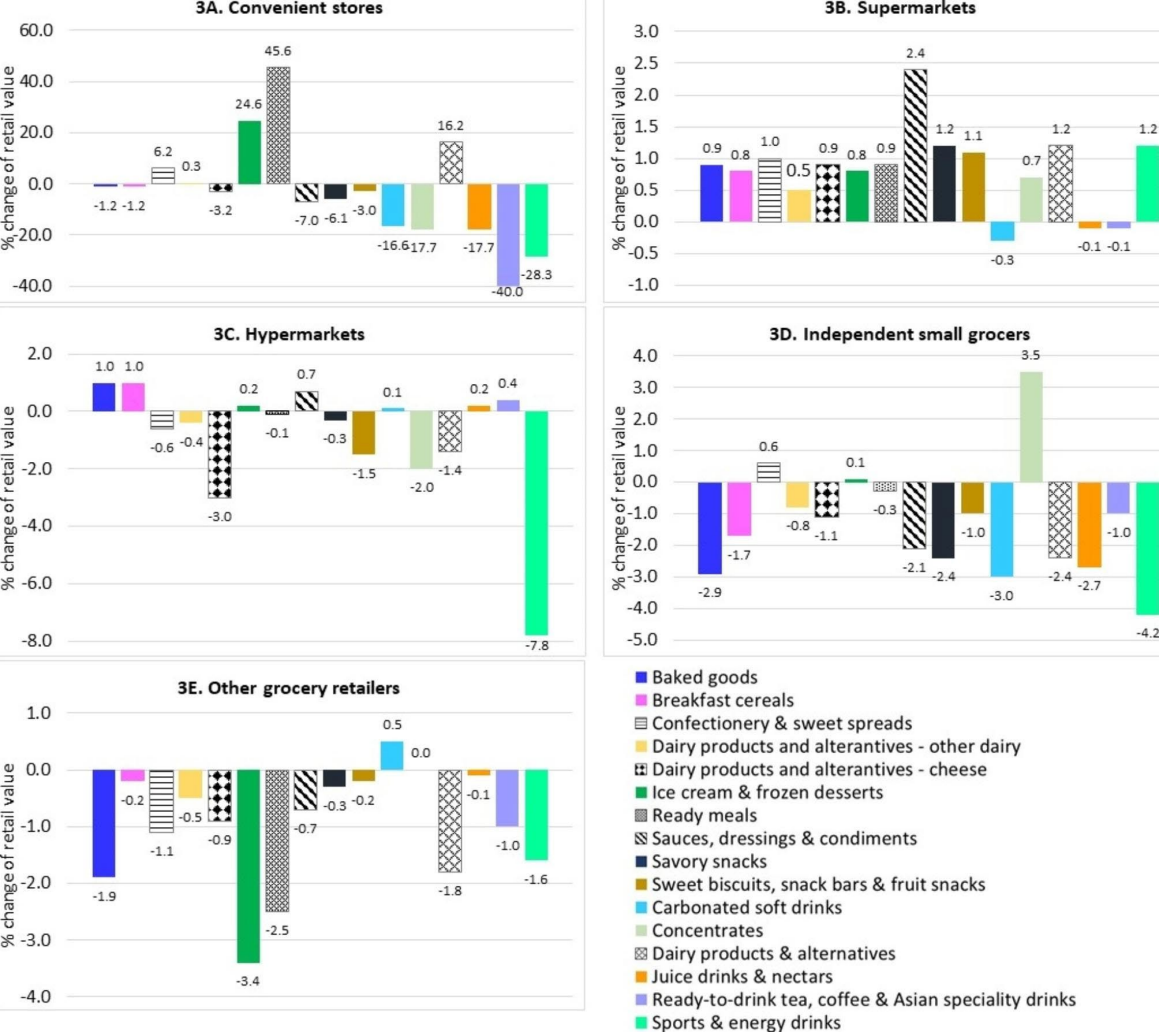
Percent change in retail value of UPF categories from 2012 to 2021 by retail format - (**A**) convenience stores, (**B**) supermarkets, (**C**) hypermarkets, (**D**) independent small grocers, and (**E**) other grocery retailers

Figure 3 presents the distribution and change in retail values of UPFs by retail format. Distribution of UPF occurs through a mix of modern channels (supermarkets, hypermarkets and convenience stores) and traditional channels (independent small grocers - mainly family-owned, and other grocer retailers - selling predominantly food, beverages and tobacco or a combination of these including kiosks, markets selling predominantly groceries).

The majority of UPF categories sold in supermarkets had increased sale values from 2012, except carbonated soft drinks, juice drinks & nectars, and ready-to-drink tea, coffee & Asian specialty drinks. The sauces, dressings & condiments category had the highest retail growth in supermarkets (2.4%). Large changes in retail value of certain UPFs were observed in convenience stores. UPF categories for ready meals, ice cream & frozen desserts, and dairy based beverages had the highest increased sale values at 45.6%, 24.6% and 16.2% from 2012 to 2021, respectively. The highest decreased sale values were observed in ready-to-drink tea, coffee & Asian specialty drinks (-40%), sports & energy drinks (-28.3%), and vegetable oils (-18.3%). For products sold in hypermarkets, the change ranged from − 7.8 to 2.4%. Baked goods and breakfast cereals categories had at least 1% sale value increase from 2012 to 2.4% and 1%, respectively. The highest drop in sales value was found in sports & energy drinks (-7.8%).

In small grocers and other grocery retailers, sales dropped in most UPF categories, and only a few had sales increases. Confectionery & sweet spreads, ice cream & frozen desserts, and concentrates had sales growth in small grocers, at 0.6%, 0.1% and 3.5%, respectively. Carbonated soft drinks were the only products sold in other grocery retailers with an increase in sales (0.5%).

### Consumer expenditure on UPF

**Fig. 4:**
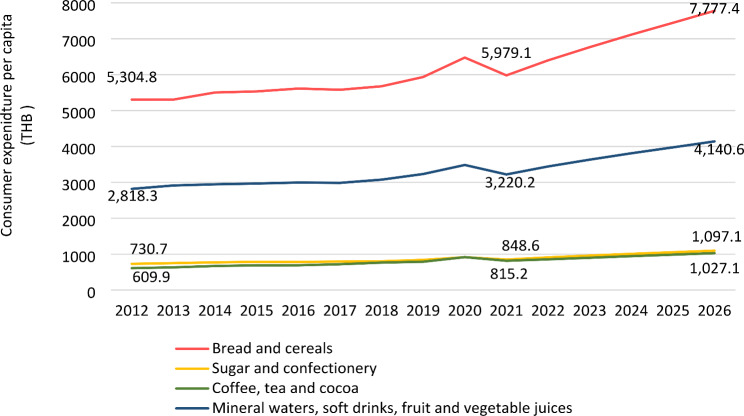
Consumer expenditure per capita (THB) of UPFs in Thailand, 2012–2021, and projections for 2021 to 2026

Overall, growth of Thai consumer expenditure per capita on UPFs in four categories (bread and cereals; sugar and confectionery; coffee, tea and cocoa; and mineral waters, soft drinks, fruit and vegetable juices) increased from 2012 to 2020, and dropped in 2021 (Fig. 4). Thai consumers spent most on bread and cereals each year (5,304.8 THB in 2012 and 5,979.1 THB in 2021). However, coffee, tea and cocoa products had the highest consumer spending growth with 33.7% increase (from 609.9 THB in 2012 to 815.2 THB in 2021).

The consumer expenditure per capita in all studied categories is forecast to grow between 26 and 30% from 2021 to 2026. Consumer spending on bread and cereals is expected to have the highest growth among four categories, at 30% (from 5,979.1 THB in 2021 to 7,777.4 THB in 2026). Spending on coffee, tea and cocoa was the only category which is forecast to have a lower percent growth (26%), compared with percent growth from 2012 to 2021 (33.7%). Meanwhile, spending on other categories is expected to have higher growth, compared with the percent growth of the previous ten years.

### Nutritional quality of UPF

A total of 17,414 packaged products identified in the Thai market were downloaded from the Mintel GNPD. Of these, 16,233 items could be evaluated using WHO SEA criteria. Of these, 12,855 items were classified as UPF. Categorising by WHO SEA food category, the food categories that had the highest percentage of UPFs were confectionery (1,391 items or 94.7% of the total sample in this category), followed by composite foods (1,376 items or 91.5%), fine bakery wares (2,559 items or 88.7%), beverages (3,151 items or 78.6%), and ready-to-eat savouries (1,006 items or 77.1%) (Table 3).

**Table 3:** Number (%) of UPF items in this study by WHO SEA categories

Categories (n) (N = 16,233)	Number of UPF items (%) (N = 12,855)
1. Confectionery (n = 1,469)	1,391 (94.7)
2. Fine bakery wares (n = 2,886)	2,559 (88.7)
3. **Bread and ordinary bakery wares (n = 344)**	**228 (66.3)**
4. **Cereals (n = 572)**	**419 (73.3)**
5. **Ready-to-eat savories (n = 1,305)**	**1,006 (77.1)**
(a) Potato, cereal or starch-based (from roots, tuber, or legumes) and animal based (from skin) (n = 950)	808 (85.1)
(b) Processed nuts (n = 197)	67 (34.0)
(c) Fish-based (n = 158)	131 (82.9)
6. **Beverages (n = 4,014)**	**3,151 (78.6)**
(a) Juices (n = 1,472)	1,052 (71.5)
(b) Milk and dairy based drinks (n = 519)	405 (78.0)
(c) Water-based flavoured drink (n = 565)	553 (97.9)
(d) Coffee, coffee substitutes, tea, herbal infusions (n = 979)	788 (80.5)
(e) Cereal, grain, tree nut-based beverages (n = 479)	353 (73.7)
7. **Frozen dairy based desserts and edible ices (n = 681)**	**614 (90.2)**
8. **Curded dairy based desserts (n = 35)**	**35 (100.0)**
9. **Cheese and analogues (n = 61)**	**29 (47.5)**
10. **Composite foods (prepared foods) (n = 1,504)**	**1,376 (91.5)**
11. **Pasta and noodles and like products (n = 477)**	**421 (88.3)**
12. **Processed meat, poultry, game, fish and fish products (n = 646)**	**493 (76.3)**
(a) Processed meat, poultry and game products (n = 312)	249 (79.8)
(b) Processed fish and seafood products (n = 334)	244 (73.1)
13. **Processed fruits and vegetables (n = 1,105)**	**438 (39.6)**
14. **Solid-form soybean products (n = 15)**	**1 (6.7)**
15. **Sauces dips, and dressings (n = 1,119)**	**694 (62.0)**

**Fig. 5:**
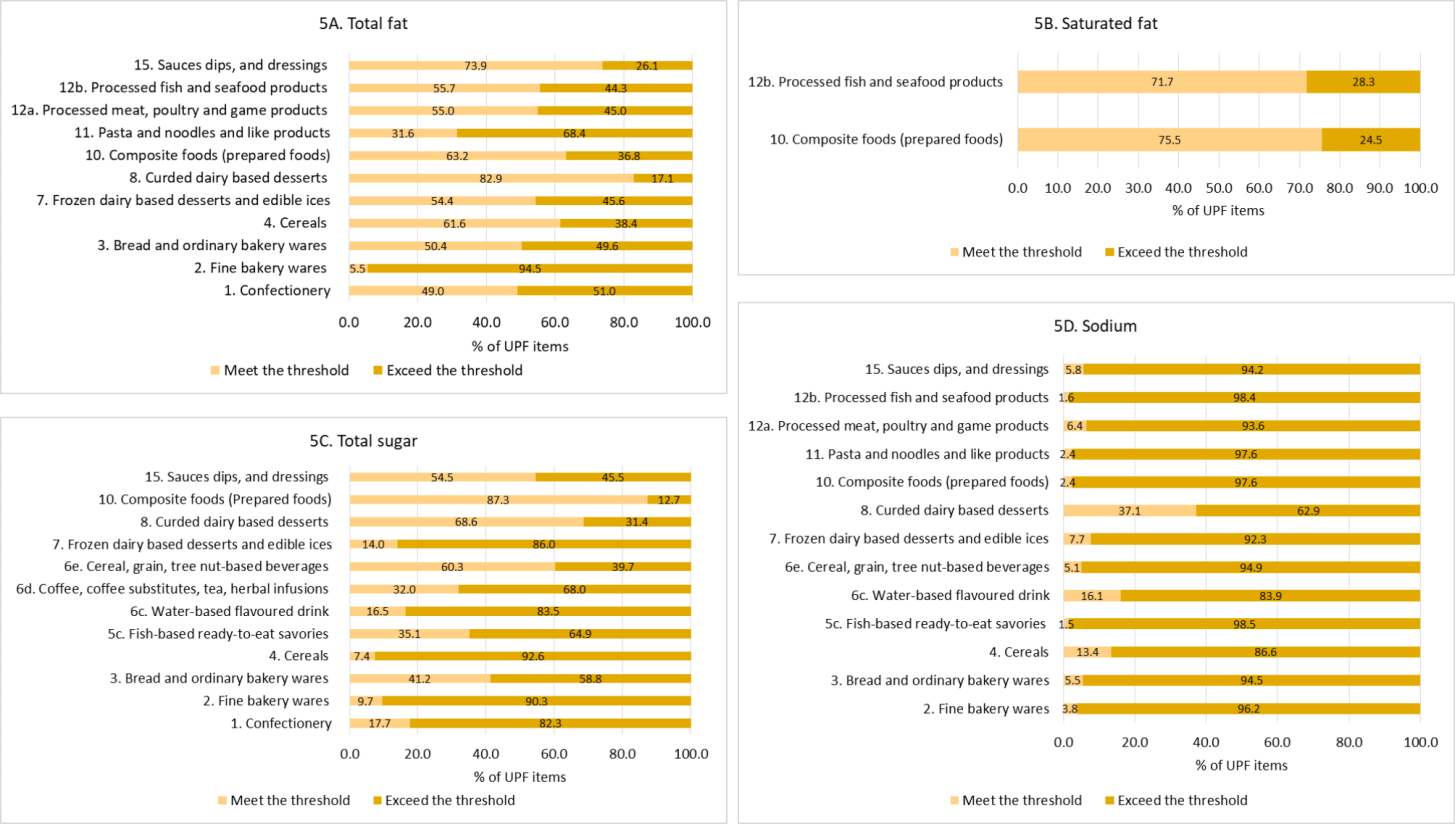
Percentage of UPF items which are evaluated based on thresholds of total fat (**A**), saturated fat (**B**), total sugars (**C**) and sodium (**D**), by food categories

Figure 5 presents the nutrition quality of UPFs in each major nutrient. More than 50% of all UPF items evaluated in this analysis exceeded the recommended levels for total fats, total sugars or sodium, and 25% exceeded the recommended level for saturated fat.

#### Total fat

Figure 5 A shows the percentage of UPF items by categories which were evaluated based on the threshold for total fat (Table 1). Overall, only 40.7% of the 8,230 UPF products evaluated met the threshold, and 59.3% contained excessive total fat content. UPFs in the following categories had more than half of the items which exceed the threshold amount of total fats: fine bakery wares (94.5%), pasta and noodles and like products (68.4%), and confectionary (51.0%).

#### Saturated fat

Figure 5B shows the percentage of UPF products by categories which were evaluated based on the threshold for saturated fat (Table 1). The WHO SEA model sets the saturated fat threshold for only the two food categories shown. Of the total of 1,620 items evaluated, the majority met the threshold and only a quarter (24.8%) failed to meet it. Processed fish and seafood products had the highest percentage of items with excessive saturated fat (28.3%), followed by composite foods (24.5%).

#### Total sugars

Figure 5 C shows the percentage of UPF products by categories which were evaluated based on the total sugar threshold (Table 1). Ten UPF categories (with total 9,140 items) were eligible for evaluation. Of the total items, 68.2% contained excessive total sugars. The UPF categories and subcategories which had over 80% of items with excessive total sugars were cereals (92.6%), fine bakery wares (90.3%), frozen dairy-based desserts and edible ices (86.0%), water-based flavoured drink (83.5%), and confectionary (82.3%). The composite foods category had the least number of items which did not meet the threshold (12.7%).

#### Sodium

Figure 5D shows the percentage of UPF items by categories which were evaluated based on the threshold sodium (Table 1). A total of 7,886 UPF items from 11 categories were evaluated. Almost all the UPF items (94.8%) contained excessive sodium content, and only 5.2% met the threshold. The top three categories or subcategories with the highest percentage of items with excessive sodium content were fish-based ready-to-eat savories (98.5%), processed fish and seafood products (98.4%), and composite foods (97.6%). Although a category in curded dairy-based desserts had the lowest percentage of items with excessive sodium, the percentage is still more than half of the total items in this category (62.9%).

## Discussion

This study aimed to analyse the national trends in retail sales of UPFs, consumer expenditure on UPFs and the nutritional quality of UPFs sold in Thailand. There are three major findings in relation to this analysis. First, the sale volumes and values of UPFs from retail outlets in Thailand has increased in the past ten years and is predicted to continue to grow in the next ten years, especially the sales growth of ready meals and flavoured and functional drinks. This is consistent with results of other studies elsewhere in Asia which found the rapid growth of UPFs [14]. This may be explained by industralisation and globalisation of food supply chains worldwide as a result of transnational food industry growth and trade liberalisation [25]. This includes more competition for a market share of food and agricultural products through the entry of transnational food and beverage companies or investors into the food systems in a host country. It corresponds to evidence of the growing sales of UPFs worldwide that is linked with technical developments, significant financial investment, and corporate political activities of food industry [26]. Furthermore, this may have been impacted by government policies and actions related to investment that focused primarily on promoting Thailand to become an economically developed country [27]. The Thailand Board of Investment Office, for example, has placed productivity and economic growth of the country as the primary purpose of its investment policies [28]. The policies focused on promoting technological innovation in food production and processing and attracting foreign direct investment into the food manufacturing sector. The government is aiming for Thailand to become a regional economic centre through a supply chain hub for major industries. These policies could be a driving force of transforming the country’s food supply chain depending on how farmers, food and agricultural suppliers, food processing industries and food retailers respond.

Although SSBs was one of the most commonly consumed food groups among Thai people [16], the sales data showed a decline in sales volume of SSBs, in particular, RTD tea and coffee & Asian specialty drinks in the past ten years with the least growth of sales values, compared with other UPF categories. However, the sales (both volumes and values) of the RTD tea, coffee & Asian specialty drinks, together with carbonated soft drinks, were the only UPF categories which were projected to have a higher growth rate (up to ten times higher) than that observed over the past 10 years. Perhaps this can be at least partly explained by the implementation of a national SSB taxation policy based on sugar content in Thailand. The taxation came into force in 16 September 2017, and was implemented by the Excise Department [29]. SSB items which contain 6 g or higher of sugar per 100 ml are taxed at a higher rate. The tax rate is expected to increase every two years until September 2023 when it reaches at 20% increase in SSB retail prices. The third phase was expected to be actioned in October 2021. However, the negative impact of the COVID-19 pandemic on growth of national economies and businesses postponed the third-phase taxation. A delay in taxation can result in a trend reversal in SSB demand increase especially in consumption. There is evidence elsewhere confirming potential impact of at least 20% tax on SSB retail sales that can result in reducing SSB consumption and calorie intake and obesity and diet-related NCDs [30–33]. Accordingly, this suggests the Thai government must strictly implement the taxation as planned.

The second finding relates to changes in the retail landscape in Thailand and that the modern retail store is one major source of UPF purchases for Thai people. The Thai retail sector has become increasingly dominated by large scale modern stores with extensive branch networks to expand their consumer base [34]. Modern wholesale and retail traders’ share of the market almost tripled from 2001 when it was 25–61% in 2014. In 2019, the modern trade sector was ranked second in generating GDP (16.5%) after the manufacturing sector (25.3% of GDP). The modern retail traders have managed to adopt a range of innovations especially distribution management and marketing strategies for their products, and thus stimulate consumer demand and continue to replace the use of traditional fresh and informal retail outlets [35]. The growth of the modern retail sector was influenced by government policies for economic development under the Thailand 4.0 policy which promotes foreign direct investors with advanced technology to invest in retail businesses [36].

Changes to retail food environments such as the increased number of modern retailers e.g., convenience stores, supermarkets and hypermarkets, and use of various marketing strategies of less healthy, pre-packaged foods have contributed to less healthy diets and health outcomes [35]. There is consistent evidence on the link between the production, advertising and promotion of UPFs and an increased risk of obesity and NCDs [35, 37–40]. This suggests a need for public health policies in regulating the marketing and availability of UPFs to limit consumer’s access to these products.

The third finding is that more than half of the UPFs sold in Thailand exceeded the amounts of at least one of the nutrients, when consumed in excess, can be detrimental to population health (fat, saturated fat, sugars, sodium) according to the criteria specified in the WHO SEA Nutrient Profile Model. Almost all (94.3%) of UPFs exceeded the sodium cut-off. These findings are consistent with a previous assessment elsewhere which found that almost all of UPFs sold in Latin American countries contained excess amounts of at least one of the nutrients [41]. This was not surprising as these nutrients are often ingredients used in UPFs to provide certain sensory effects. These ingredients are often food substances of no or rare culinary use (such as fructose, high-fructose corn syrup, fruit juice concentrates and modified oils) that are combined with “cosmetic” additives (such as flavour enhancers, colours, emulsifiers, carbonating, gelling and glazing agents), in order to make the final product visually attractive and palatable [9]. Although it is acknowledged that excessive content of certain nutrients is not the only mechanism by which UPFs are linked to adverse health outcomes, and thus monitoring of the entire UPF category regardless of nutrient content is necessary.

To the best of our knowledge, this is the first profile analysis of supply and demand for UPFs in Thailand and Southeast Asia. No systematic study has estimated the relative importance of UPF sales in the country. The present study addressed this important information gap in order to give a picture of the extent of proliferation of UPFs in Thailand, which provides a baseline for future monitoring and the rationale for food and nutrition policies aimed at restricting the sales of UPFs as well as addressing one crucial aspect of the burden of obesity and diet-related NCDs.

The proliferation and purchasing of UPFs has been observed in many countries around the world and is a powerful marker of the nutrition transition. The study has demonstrated a robust approach for effectively monitoring trends in retail sales of UPFs, consumer expenditure on UPFs and nutritional quality of UPFs sold in Thailand. The databases and analytical approaches used in this study could provide a template for monitoring UPF sales and consumer purchasing in other low- and middle-income countries around the world.

The combined use of the NOVA classification scheme and the WHO SEA Nutrient Profile Model allowed for a more holistic assessment of the healthfulness of the foods contributing to sales, consumer expenditure and consumption in Thailand [42]. It is clear that the traditional diets of Thai people which depended largely on their surroundings [43] is being undermined. Thai dishes were traditionally prepared based on local unprocessed or minimally processed ingredients such as vegetables as the main ingredients and fish as the main source of protein, with simple cooking methods. The dishes were also served in bite-size pieces. Thailand used to be described as a land of abundance where “in the water there are fish, in the field there is rice [44].”

There are some limitations of this study. The study relied on the data from the Euromonitor and Mintel GNPD databases that retrieve product information mainly from registered modern retailers, and thus may miss some locally made or sold products in local and traditional stores. The databases also retrieved the information from different sources and categorised products differently, and thus are not perfectly matched across categories. The Euromonitor also lacked subcategories and ingredient information that made accurate UPF classification particularly difficult, and resulted in some food categories being included or excluded, potentially leading to some misclassifications. Furthermore, the sales and expenditure data were aggregated, and so it wasn’t possible, for instance, to examine sales and consumer expenditures on UPFs across different sociodemographic groups within Thailand. In addition, in the expenditure data some non-UPF and UPF products were aggregated and presented together. However, the strength of using consumer expenditure data is that it can be used as a proxy for actual consumption. Moreover, the data from these sources can complement each other as the Euromonitor provides the UPF sales and consumer expenditure data while the Mintel GNPD provides characteristics and ingredient description of UPF. Moreover, the databases are the largest data sources that provide detailed information about characteristics and ingredient descriptions of food and beverage items marketed in many countries, making up nearly a quarter of world’s countries, including Thailand. They are also widely used for research studies in public health policy such as in nutrition, tobacco and alcohol at a country level and for cross-country comparison, and thus are likely to be reliable.

### Policy implications

The study highlights a number of policy implications. First, the findings suggest future research is needed to examine the effects of policy actions, especially regulatory measures, such as taxation and regulation to in-store marketing and availability of UPFs that can effectively reduce the demand for UPFs. As there is not yet any clear evidence on effectiveness of these policies for reducing UPFs in Thailand, lessons from other countries can be useful in guiding future implementation. For example, lessons learned from studies on Mexico and South Africa’s SSB taxes show those of lower socio-economic status who are likely to have a higher burden of obesity and diet-related NCDs were more likely to reduce their SSB purchases as affected the tax [45–48]. Chile implemented UPF marketing ban that results in decrease in exposure to advertisement of UPFs in children and adolescents [45, 49]. In addition to this, to enhance effectiveness in Thailand, this regulatory measure should be combined with the provision of market incentives to make unprocessed and minimally processed food, and freshly prepared dishes and meals, more available, accessible and affordable. The combined regulatory and non-regulatory measures may also allow the traditional grocers to increasingly usurp the position of modern traders, and thus lead to improvement in population diets. It is also important to develop an ongoing monitoring and assessment system for sales, consumer purchases and nutritional quality of food and beverage products in Thailand to inform the implementation of policies in support of healthy and sustainable eating patterns.

## Conclusions

The study provides valuable data to understand the role of UPF as a driver of the nutrition transition in Thailand. These data have two valuable applications: provision of evidence for informing future policy activities, and contribution to the setting up of an ongoing monitoring system. The study also provides additional data to the UPF studies all showing rapid growth in UPFs around the world – there is relatively little data available from Asia in general and Southeast Asia in particular. The data shows the link of Thailand’s nutrition transition with the globalisation of industrial food systems that are facilitated by global trade and investment—into both processing, marketing, and retailing of transnational food corporations.

The findings, particularly in relation to the UPF category and, suggest that taxation has been an effective measure in reducing sales of SSBs, and that regulatory policy measures such as taxation and in-store marketing regulation should be applied to reduce the demand for, availability of and access to UPFs to improve diets and health of the Thai population. The regulatory measures should be taken together with the provision of market incentives to make unprocessed and minimally processed food, and freshly prepared dishes and meals, more available, accessible and affordable. Monitoring food environment changes is necessary to contribute to policy decision making and to determining the effectiveness of current policy actions. The approach used in this study could fulfill this monitoring role for Thailand and other similarly developed countries within Asia and the Pacific.

### Electronic supplementary material

Below is the link to the electronic supplementary material.

**Table A1**. Criteria for NOVA classification

**Table A2**. Category definition for per capita sales volumes analysis according to Euromonitor Database

**Table A3**. Category definition for consumer expenditure analysis according to Euromonitor Database

## Data Availability

The datasets generated and/or analysed during the current study are not publicly available. Access to data is available from the corresponding author on reasonable request.

## Abbreviations

FAO: Food and Agriculture Organization of the United Nations
GNPD: Mintel Global New Products Database
NCD: Non-communicable disease
RTD: Ready-to-drink
SEA: South East Asia
SSB: Sugar sweetened beverage
THB: Thai baht
UPF: Ultra-processed food
WHO: World Health Organization
