# Supplementary material for: Profiling ultra-processed foods in Thailand: sales trend, consumer expenditure and nutritional quality
Source: Global Health. 2023 Aug 31;19:64. doi: 10.1186/s12992-023-00966-1 (PMC10472697; doi:10.1186/s12992-023-00966-1)
Supplement: Supplementary file 1 — Table A1. Criteria for NOVA classification [file 12992_2023_966_MOESM1_ESM.docx]

**Appendix A.**

**Table A1. Criteria for NOVA classification**

(Source: Monteiro, C. A., Cannon, G., Lawrence, M., Costa Louzada, M. L., & Pereira Machado, P. (2019). Ultra-processed foods, diet quality, and health using the NOVA classification system. https://www.fao.org/3/ca5644en/ca5644en.pdf)

| **Group** | **Concept** | **Examples** |
| --- | --- | --- |
| **Group 1** Unprocessed and minimally processed foods | Foods unaltered or altered by processes such as removing inedible parts, drying, grinding, cooking, pasteurization, freezing, or non-alcoholic fermentation. No substances are added. Processing aims to increase food stability and enable easier or more diverse preparation. | fresh or frozen fruit and vegetables, fresh or frozen meats and seafood, plain dried pasta (no salt), flours, rice, oats, other grains, pasteurized milk, yoghurts with no added sugar or flavours, dry pulses, fruit or vegetable juices, plain seaweed, fruit purees, dried fruit, nuts, seeds, herbs and spice (including dried and powdered), powdered or dried forms of any Group 1 foods, cocoa, coffee beans |
| **Group 2**  Processed culinary ingredients | Substances obtained directly from Group 1 foods or from nature, created by industrial processes such as pressing, centrifuging, refining, extracting or mining. Processing aims to create products to be used in preparation, seasoning and cooking of Group 1 foods | salt, butter, vegetable oils, olive oils  seed and nut oils, lard, other fats, sugar (sucrose, cane sugar), brown sugar, molasses, honey, maple syrup, sugar syrup (cane sugar), vinegar, plain starches - not modified starches (corn starch, tapioca starch), raising agents (e.g. bicarb soda), coconut milk or cream (including those made with coconut extract), mineral salts |
| **Group 3**  Processed foods | Products made by adding edible substances from Group 2 to Group 1 foods using preservation methods such as non- alcoholic fermentation, canning, or bottling. Processing aims to increase stability and durability of Group 1 foods and to make them more enjoyable. | canned vegetables (with added salt), canned fruit in sugar syrup, canned pulses (with added salt), cheese, cured meats (salami/ham), shrimp paste (assumed to be group 3 when no secondary ingredient information), fish sauce (assumed to be group 3 when no secondary ingredient information), yoghurt with added sugar, a mixed meal with vegetable oils and salt |
| **Group 4**  Ultra-processed foods | Formulations of low-cost sub- stances derived from Group 1 foods with little to no whole foods; always contain edible substances not used in home kitchens (e.g., protein isolates) and/or cosmetic additives (e.g., flavors, colors, emulsifiers). Processing involves multiple steps and industries and aims to create products liable to replace all other NOVA groups. | carbonated soft drinks, sweet or savory packaged snacks; chocolate, candies (confectionery), ice cream; mass-produced packaged breads and buns; margarines and other spreads; cookies (biscuits), pastries, cakes and cake mixes; breakfast ‘cereals’; pre-prepared pies and pasta and pizza dishes; poultry and fish ‘nuggets’ and ‘sticks’, sausages, burgers, hot dogs and other reconstituted meat products; powdered and packaged ‘instant’ soups, noodles and desserts; and many other products |
