# Supplementary material for: Profiling ultra-processed foods in Thailand: sales trend, consumer expenditure and nutritional quality
Source: Global Health. 2023 Aug 31;19:64. doi: 10.1186/s12992-023-00966-1 (PMC10472697; doi:10.1186/s12992-023-00966-1)
Supplement: Supplementary file 2 — Table A2. Category definition for per capita sales volumes analysis according to Euromonitor Database [file 12992_2023_966_MOESM2_ESM.docx]

**Appendix A.**

**Table A2. Category definition for per capita sales volumes analysis according to Euromonitor Database**

| **Category** | **Definition** |
| --- | --- |
| **Ultra-processed foods** |  |
| Baked goods | The aggregation of bread, pastries, dessert mixes, frozen baked goods and cakes.  Note: Baked goods from in-store bakeries are classified under unpackaged/artisanal, not packaged/industrial. While they may be finished on-site, they are often prepared, then frozen or par-baked, at other locations. Such production models are very important for supermarket in-store bakeries, which have in the past been used to drive traffic and fill stores with appetising aromas, but for which the labour resources required to run a full-service scratch bakery are not always available. |
| Breakfast cereals | The aggregation of ready-to-eat and hot cereals. |
| Confectionery | The aggregation of chocolate confectionery, sugar confectionery and gum.  Chocolate confectionery: The aggregation of tablets, countlines, bagged selflines/softlines, boxed assortments, seasonal chocolate, chocolate with toys, alfajores and other chocolate confectionery. Note that chocolate overtly positioned for baking/cooking purposes is excluded from Euromonitor International's confectionery coverage.  Sugar confectionery: The aggregation of mints, boiled sweets, pastilles, gums, jellies and chews, toffees, caramels, nougat, medicated confectionery, lollipops, liquorice and other sugar confectionery.  Gum: The aggregation of chewing and bubble gum.  Note: Retail sales measurements are confined to packaged sales. However, exceptions are made to seasonal chocolate, where unpackaged/artisanal sales are included. Pick ‘n’ mix sales are also included. Finally sales from chocolatiers, typically displayed loose and later packed (usually in boxes) are also included. |
| Sweet spreads | The aggregation of jams and preserves, honey, chocolate spreads, and nut and seed based spreads. |
| Dairy products & alternatives | The aggregation of diary and plant based diary. It excludes drinking diary products.  Dairy: This is the aggregation of butter and spreads, cheese, yoghurt products and other dairy. Non-dairy alternatives are included within plant-based dairy.  Plant-based dairy: This is the aggregation of plant-based milk, plant-based yoghurt and plant-based cheese. Please note that plant-based dairy products that are not milk, yoghurt or cheese are tracked under their core dairy categories (e.g. powder milk, margarine and spreads, coffee whiteners etc.) |
| Processed fruit and vegetables | The aggregation of processed shelf stable fruit and vegetables and processed frozen fruit and vegetables. |
| Frozen processed potatoes | The aggregation of frozen processed potatoes. |
| Ice cream & frozen desserts | The aggregation of frozen yoghurt, impulse ice cream, unpackaged ice cream and take-home ice cream. Frozen desserts is included in the project staples. Includes: Non-dairy ice creams (for example, soy or rice-based products, as well as any other dairy-alternative ice creams), should be tracked alongside dairy ice cream in the relevant product subcategory. Rice and soy can be used as dairy substitutes in the manufacture of ice cream, but the product is still equivalent in terms of positioning/marketing and consumer target to standard dairy ice cream. |
| Instant noodles | The aggregation of noodles sold in a precooked and dried block with flavoring powder and/or seasoning oil. |
| Processed meat & seafood | The aggregation of processed meat, processed seafood and meat and seafood substitutes. |
| Ready meals | The aggregation of shelf stable, frozen, dried, chilled ready meals, dinner mixes, frozen pizza, chilled pizza and prepared salads.  Note: Ready meals are products that have had recipe ''skills'' added to them by the manufacturer, resulting in a high degree of readiness, completion and convenience. Ready meals are generally accepted to be complete meals that require few or no extra ingredients, however, in the case of canned/preserved ready meals, the term also encompasses meal ''centres’; for dinner mixes, the term encompasses part meals. Some ready meals may require cooking; others may simply need reheating, prior to serving. |
| Sauces, dressings & condiments | The aggregation of cooking ingredients, dips, pickled products, table sauces, tomato pastes and purées, yeast-based spreads, and other sauces, dressings and condiments. It excludes table salt, baking ingredients, vinegar, and cooking cream. |
| Savoury snacks | The aggregation of chips/crisps, extruded snacks, tortilla/corn chips, popcorn, pretzels, nuts and other sweet and savoury snacks |
| Sweet biscuits, snack bars & fruit snacks | The aggregation of biscuits and snack bars. |
| **Ultra-processed beverages** |  |
| Carbonated soft drinks | Sweetened, non-alcoholic drinks containing carbon dioxide are included here. All carbonated products containing fruit juice (“sparkling juices”) are included here, unless they are tea-based (these are included in carbonated RTD tea) or carbonated Energy drinks, which are included in Energy Drinks. Carbonated bottled water is also excluded. Carbonates are an aggregation of cola carbonates and non-cola carbonates, whether regular or low calorie. Euromonitor International includes both naturally and artificially-sweetened carbonates. |
| Soft drink concentrates | The aggregation of liquid concentrates and powder concentrates. |
| Dairy products & alternatives | The aggregation of drinking diary products. It includes drinking milk products, yoghurt drink products, sour milk products and plant-based milk. |
| Functional & flavoured water | Functional water: Water which offers additional health and other functional benefits. It is enhanced by supplemental ingredients, which are also known as aquaceuticals, such as vitamins, minerals, acids, herbs, raw fruits or vegetables.  Flavored waters: Waters that have had natural and/or artificial flavors added in order to enhance the taste of the water. Functional waters maybe flavored as well but also include vitamins, minerals, or infusions that are meant to provide increased health benefits to the consumer. |
| Juice drinks & nectars | This category covers all still packaged juice obtained from fruits or vegetables by mechanical processes, reconstituted or fresh, often including pulp or fruit/vegetable puree. All unpackaged juices are excluded. Only still drinks are included here. Carbonated varieties are included non-cola carbonates. Juice-flavoured milk drinks and fruit shakes which are primarily milk are excluded–these are instead tracked in Packaged Foods Dairy. However, if the juice component is greater, the product is to be excluded from Packaged Foods Dairy coverage and tracked under the relevant category (based on % juice content) within Soft Drinks juice. This sector is the aggregation of 100% juice, nectars (25-99% juice content), juice drinks (up to 24% juice content), and coconut & other plant waters. |
| RTD tea, coffee & Asian specialty drinks | Ready-to-drink (RTD) coffee: This category includes packaged ready-to-drink coffee, consumed either hot or cold, made using a base of either brewed coffee or coffee extract. Excludes all coffee flavoured milk drinks that primarily target children, or where coffee is one of a number of flavours within the brand range. Leading brands in off-trade volume include Georgia, Nescafé and Suntory Boss.  RTD tea: This category includes all packaged products based on brewed tea or tea extract. May be sweetened or unsweetened, carbonated or still, with a wide variety of different flavourings. May contain juice.  Asian speciality drinks: This category includes all traditional Asian drinks not included in RTD tea or juice drinks, including products such as Bandung (rose syrup with milk), bird’s nest, tamarind juice, ginger, lemongrass, roselle, zalaka, jelly drinks including grass jelly (cincau), sugar cane, and vinegar drinks. Lactic acid drinks, such as Calpis, are included here. Drinks containing a limited amount of yogurt (generally 3% or less) such as Bikkle, are included here, though drinking yogurts such as Yakult are excluded. While both products are highly popular in markets like Japan, drinking yogurts will contain mostly yogurt with a very short shelf life (two weeks or less), while yogurt drinks will contain less than 3% dairy and remain on the shelves for up to 9 months. All nut or pulse-based products, such as peanut milk, almond juice, or soy drinks are tracked in Non-Dairy Milk alternatives in Passport Packaged Food. |
| Sports & energy drinks | Sports drinks: The choice of sports drink usually depends on the provision of fluids, carbohydrates or both. Included into this subsector are isotonic, hypotonic and hypertonic sports drinks. Isotonic are products that replace lost body fluids, electrolytes (sodium, potassium and chlorides) and glucose in similar concentrations to existing body fluid without causing either swelling or shrinkage of cells. These products usually contain about 5-8% carbohydrate and are intended to be consumed during exercise and/or heat exposure. Hypotonic this product is a weaker solution than your body fluid. These drinks contain less carbohydrate and therefore have lower osmolality (fewer dissolved particles than blood). These drinks help the body to speed up water absorption and are best used when you need urgent fluid replacement, as in after exercise. These drinks are not the best for energy replacement. Hypertonic - this drink is a stronger solution than your body fluid. These drinks are designed to replace and maintain energy levels during exercise of at least one hour. They are absorbed slowly and therefore are not appropriate for fluid replacement. Leading brands in off-trade volume include Gatorade, Powerade and Aquarius.  Energy drinks: These are functional drinks designed to boost energy levels. Often lightly carbonated, these contain high levels of caffeine and a number of added water-soluble vitamins, most often a selection of B vitamins including niacin, pantothenic acid, vitamin B6, and vitamin B12. Ingredients can also include amino acids such as taurine and glucuronolactone, as well as herbal products such as guarana and ginseng. Can be carbonated or still. Leading brands in off-trade volume include Red Bull, Monster and RockStar. Glucose-based energy beverages such as Lucozade Energy are included here. |
