# Supplementary material for: Profiling ultra-processed foods in Thailand: sales trend, consumer expenditure and nutritional quality
Source: Global Health. 2023 Aug 31;19:64. doi: 10.1186/s12992-023-00966-1 (PMC10472697; doi:10.1186/s12992-023-00966-1)
Supplement: Supplementary file 3 — Table A3. Category definition for consumer expenditure analysis according to Euromonitor Database [file 12992_2023_966_MOESM3_ESM.docx]

**Appendix A.**

**Table A3. Category definition for consumer expenditure analysis according to Euromonitor Database**

| **Category** | **Definition** |
| --- | --- |
| **Ultra-processed foods** |  |
| Bread and cereals | The aggregation of rice in all forms, maize, wheat, barley, oats, rye and other cereals in the form of grain, flour or meal, bread and other bakery products (crispbread, rusks, toasted bread, biscuits, gingerbread, wafers, waffles, crumpets, muffins, croissants, cakes, tarts, pies, quiches, pizzas, etc.), mixes and doughs for the preparation of bakery products, pasta products in all forms, couscous, cereal preparations (cornflakes, oat flakes, etc.) and other cereal products (malt, malt flour, malt extract, potato starch, tapioca, sago and other starches), farinaceous-based products prepared with meat, fish, seafood, cheese, vegetables or fruit. This category excludes meat pies (consumer expenditure on meat), fish pies (consumer expenditure on fish and seafood), sweet corn (consumer expenditure on vegetables). |
| Sugar and confectionery | The aggregation of cane or beet sugar, unrefined or refined, powdered, crystallized or in lumps, jams, marmalades, compotes, jellies, fruit purées and pastes, natural and artificial honey, maple syrup, molasses and parts of plants preserved in sugar, chocolate in bars or slabs, chewing gum, sweets, toffees, pastilles and other confectionery products, cocoa-based foods and cocoa-based dessert preparations, edible ice, ice cream and sorbet, artificial sugar substitutes. This category excludes cocoa and chocolate-based powder (consumer expenditure on coffee, tea and cocoa). |
| **Ultra-processed beverages** |  |
| Coffee, tea and cocoa | The aggregation of coffee, whether or not decaffeinated, roasted or ground, including instant coffee, tea, maté and other plant products for infusions, cocoa, whether or not sweetened, and chocolate-based powder, cocoa-based beverage preparations, coffee and tea substitutes, extracts and essences of coffee and tea. This category excludes chocolate in bars or slabs, cocoa-based food and cocoa-based dessert preparations (consumer expenditure on sugar and confectionery). |
| Mineral waters, soft drinks, fruit and vegetable juices | The aggregation of mineral or spring waters, all drinking water sold in containers, soft drinks such as sodas, lemonades and colas, fruit and vegetable juices, syrups and concentrates for the preparation of beverages. This category excludes non-alcoholic beverages which are generally alcoholic such as non-alcoholic beer (consumer expenditure on alcoholic drinks). |
